# From risk to resilience: a narrative overview of modifiable factors influencing depression in children with autism spectrum disorder (part I)

**DOI:** 10.3389/fpsyt.2026.1859969

**Published:** 2026-08-05

**Authors:** Emanuela Elena Mihai, Ileana Ciobanu, Matei Teodorescu, Andreea Georgiana Marin, Koushik Maharatna, Mihai Berteanu

**Affiliations:** 1 Department of Physical and Rehabilitation Medicine, Carol Davila University of Medicine and Pharmacy Bucharest, Bucharest, Romania; 2 Elias University Emergency Hospital, Bucharest, Romania; 3 School of Electronics and Computer Science, University of Southampton, Southampton, United Kingdom

**Keywords:** autism spectrum disorder (ASD), children and adolescents, depression, mental health, modifiable factors for depression, protective factors, risk factors

## Abstract

**Background:**

Autism Spectrum Disorder (ASD) is a complex neurodevelopmental condition frequently associated with internalizing disorders, particularly depression, which significantly impacts functioning and quality of life (QoL) in children and adolescents.

**Objective:**

This paper represents the first part of a two-part structured narrative overview examining internalizing psychopathology in children and adolescents with ASD. Given the high co-occurrence and shared neurobiological, psychological, and environmental mechanisms linking depression and anxiety, the multidisciplinary research team elected to develop two complementary overviews to allow a more comprehensive and clinically meaningful synthesis of the evidence. The present review specifically aims to identify and synthesize current evidence on modifiable risk and protective factors influencing depression in children and adolescents with ASD.

**Methods:**

A structured narrative overview was conducted, integrating evidence from systematic reviews, meta-analyses, and robust clinical studies. Studies were selected based on relevance and methodological quality, and findings were synthesized across biological, psychosocial, and lifestyle domains.

**Results:**

Growing body of evidence indicates that depression in ASD is influenced by a complex interplay of genetic vulnerability, sensory and emotional dysregulation, social stressors, and environmental factors. At the same time, modifiable protective factors—including physical activity, psychosocial interventions, digital health approaches, and family-centered care—show potential in enhancing resilience and emotional well-being. However, the evidence base remains heterogeneous and often limited by methodological constraints.

**Conclusions:**

A multidisciplinary and prevention-oriented approach that simultaneously addresses risk and strengthens protective pathways may optimize mental health outcomes and improve QoL in children and adolescents with ASD.

## 1 Introduction

Autism Spectrum Disorder (ASD) is a complex neurodevelopmental and psychiatric condition characterized by persistent deficits in social communication and interaction, alongside restricted and repetitive patterns of behavior (1, 2). Its etiology is multifactorial, reflecting a dynamic interplay between genetic susceptibility and environmental influences. Both depression and anxiety are common and clinically significant comorbidities in children and adolescents with ASD and are increasingly recognized as major contributors to functional impairment and reduced quality of life within this population (3). These conditions contribute substantially to symptom severity, functional impairment, reduced quality of life (QoL), and increased caregiver burden in children and adolescents with ASD, underscoring the need for comprehensive and multidimensional management strategies (1, 2, 4–6).

ASD management should be comprehensive to achieve positive results through multidisciplinary, biomedical, and behavioral therapies. Early diagnosis, adapted therapeutic interventions, as well as the comprehension of aggravating and protective factors, can greatly improve the outcomes (1, 2, 4–6). Therefore, understanding the role of these factors is essential for the effective management of ASD, as it may help clarify their contribution to symptom severity and overall clinical outcomes (1). To date, much of the existing literature has concentrated on identifying risk factors associated with depression in ASD, including neurobiological vulnerability, sensory dysregulation, environmental exposures, psychosocial stressors, and maladaptive coping mechanisms (1, 3). While this risk-focused framework has substantially advanced the understanding of the etiology of depression in ASD, it provides an incomplete basis for clinical decision-making if not complemented by the identification of factors that promote resilience and emotional well-being. Emerging evidence suggests that early diagnosis, timely and individualized interventions, supportive psychosocial environments, rehabilitation strategies, physical activity, and other lifestyle-related approaches may mitigate emotional distress and reduce the burden of depressive symptoms in this population (7, 8). However, these potentially protective influences have often been investigated in isolation and rarely synthesized alongside established risk pathways within an integrated conceptual framework. Within this context, interventions such as physical activity, structured exercise programs, occupational therapy, sensory integration, cognitive-behavioral therapies, and digitally supported psychosocial interventions may exert protective effects through neuroplastic, behavioral, and psychosocial mechanisms. These approaches align with contemporary multidisciplinary models of ASD care, which emphasize functional participation, emotional regulation, and long-term well-being rather than symptom control alone (9–11).

To the best of our knowledge, no previous narrative overview has comprehensively integrated both modifiable risk and protective factors influencing depression in children and adolescents with ASD from a multidisciplinary and rehabilitation-oriented perspective. Therefore, the present narrative overview was designed to provide a comprehensive synthesis of the available evidence and to support a prevention-oriented framework that may inform future research, clinical practice, and individualized interventions.

A structured narrative overview was considered the most appropriate methodology because the objective was to synthesize and critically interpret heterogeneous evidence spanning biological, psychological, environmental, and rehabilitative domains. The breadth and heterogeneity of the available evidence, encompassing systematic reviews, meta-analyses, randomized and observational studies, together with clinically relevant multidisciplinary literature, made this approach particularly suitable for integrating diverse sources of evidence into a coherent, clinically oriented framework. Unlike systematic reviews or meta-analyses, which are generally designed to answer narrowly defined questions using predefined protocols and, where appropriate, quantitative synthesis, or scoping reviews, which primarily map the breadth of available evidence, this approach enables the integration and contextualization of heterogeneous findings while identifying conceptual relationships, knowledge gaps, and implications for multidisciplinary practice.

Depression and anxiety frequently co-occur in children and adolescents with ASD and share overlapping neurobiological, psychological, and environmental determinants. This comorbidity complicates both assessment and management, as symptoms often interact and reinforce one another over time. To address this complexity while maintaining conceptual clarity, the multidisciplinary research team structured the work as a two-part narrative overview. The present paper focuses specifically on depression, whereas the complementary paper examines anxiety using the same conceptual framework and methodological approach. This design enables both an in-depth evaluation of disorder-specific evidence and an integrated understanding of internalizing psychopathology in ASD.

## 2 Materials and methods

### 2.1 Narrative review approach strategy

A structured narrative review approach was selected to facilitate the synthesis and critical interpretation of a heterogeneous body of evidence spanning biological, psychological, environmental, and rehabilitative domains. This methodology enables a broader contextualization of findings within real-world clinical and healthcare settings, supports the integration of diverse sources of evidence, and promotes a multidisciplinary, prevention-oriented perspective aimed at improving awareness of mental health disorders (MHD) and enhancing quality of life (QoL) in vulnerable populations. Consistent with these objectives, the narrative review methodology facilitated the identification of conceptual patterns, critical interpretation of heterogeneous evidence, and discussion of clinically relevant implications rather than quantitative synthesis. The review methodology was informed by recommendations for high-quality narrative reviews, including transparent reporting of the search strategy, structured evidence synthesis, and critical interpretation, as reflected in the SANRA framework (12). The review framework was developed collaboratively by the multidisciplinary research team (E.E.M., I.C., M.T., A.G.M., K.M., M.B.), whose expertise in education, research, and clinical practice informed the conceptual development and interpretation of the evidence. The conceptual framework was further refined through iterative multidisciplinary discussions informed by current evidence and clinical experience.

### 2.2 Search strategy and study selection

A structured, non-systematic literature search was performed across PubMed, PubMed Central (PMC), Scopus, and Google Scholar. In contrast to a systematic review designed to identify all eligible studies, the search was conducted iteratively to identify the most relevant and methodologically robust evidence addressing the objectives of this narrative overview. Searches were performed iteratively between April 2024 and October 2025, allowing the search strategy to be progressively refined as the review evolved. The final literature search was completed in October 2025.

The search strategy combined keywords and MeSH terms related to autism spectrum disorder (ASD), depression, depressive symptoms, risk factors, protective factors, modifiable factors, resilience, mental health, prevention, psychosocial interventions, rehabilitation, sensory regulation, lifestyle interventions, personalized medicine, and children and adolescents. Search terms were adapted to the requirements of each database to optimize retrieval. No restrictions were applied regarding country, study design, outcomes, or publication date. Only full-text articles published in English were considered eligible.

Retrieved records underwent title and abstract screening followed by full-text assessment conducted independently by four reviewers (E.E.M., I.C., M.T., and A.G.M.). Disagreements were resolved through discussion with two additional reviewers (K.M. and M.B.) until consensus was achieved. To improve coverage of the available evidence, reference lists of eligible publications were also screened to identify additional relevant studies not captured during the database searches. Consistent with the objectives of a structured narrative overview, study selection prioritized relevance to the review objectives, methodological robustness, and contribution to the conceptual framework rather than exhaustive inclusion of all available literature.

A summary of the literature identification and study selection process is presented in **Supplementary Figure 1**, while representative search strategies, search dates, and iterative refinements are provided in **Supplementary Table 1**. As this review forms Part I of a two-part narrative overview, several literature searches were conducted concomitantly with those undertaken for the companion anxiety-focused review before the evidence was synthesized separately according to the specific objectives of each overview. Because multiple iterative searches were performed across databases and additional relevant studies were identified through reference-list screening, retrieval counts should not be interpreted as representing unique records or the final number of included studies.

### 2.3 Eligibility: inclusion and exclusion criteria

#### 2.3.1 Inclusion criteria

Studies were considered eligible if they met the following criteria:

1. Population: included children and adolescents (typically aged 0–18 years) diagnosed with Autism Spectrum Disorder (ASD), with reported outcomes related to depression, depressive symptoms, or associated internalizing features. Risk factors and protective factors interacting with depression in ASD young population were included. Studies including mixed-age populations were considered if pediatric data could be clearly identified or represented a substantial proportion of the sample.
2. Study design: a broad range of study designs was included to capture the heterogeneity of the evidence base, including randomized controlled trials (RCTs), non-randomized trials (NRTs), observational studies (cohort, case–control, and cross-sectional), case series, as well as systematic reviews and meta-analyses. When systematic reviews and meta-analyses were included, evidence was synthesized at the level of their published findings rather than by re-extracting the individual primary studies they contained. Primary empirical studies were considered independently only when they were directly included in the present narrative overview, thereby minimizing duplicate interpretation of the same evidence.
3. Outcomes: studies assessing depression as a primary or secondary outcome, including clinically diagnosed depressive disorders, validated rating scales (e.g. PHQ-9), or proxy measures related to mood and emotional functioning.
4. Interventions/exposures: studies evaluating modifiable risk factors, protective factors, or therapeutic interventions (e.g., rehabilitation, psychosocial, pharmacological, sensory, lifestyle-based, or digital health approaches) associated with depressive symptoms in ASD populations.
5. Publication characteristics: peer-reviewed articles published in English with sufficient methodological detail and accessible full-text.

#### 2.3.2 Exclusion criteria

Studies were excluded based on the following criteria:

1. Population: studies focusing exclusively on adults with ASD, or on neurotypical populations without separate analysis for individuals with ASD; studies where ASD diagnosis was not clearly defined or validated.
2. Outcomes: studies not reporting depression-related outcomes or focusing solely on other psychiatric comorbidities (e.g., ADHD, anxiety) without reference to depressive symptoms and their entwined nature, as depression, ADHD, and anxiety are frequently related.
3. Publication type: editorials, opinion papers, conference abstracts without sufficient methodological detail or full data, narrative commentaries lacking empirical evidence, and single case reports with limited generalizability.
4. Methodological limitations: studies with insufficient methodological transparency, unclear outcome measures, or lacking relevance to the conceptual framework of modifiable risk and protective factors.
5. Redundancy: duplicate publications or studies with overlapping datasets, in which case the most comprehensive or recent version was retained.

Finally, evidence was extracted and synthesized from 65 papers regarding the interplay of risk and protective factors, as well as interventions and strategies for modulating depression in children and adolescents with ASD. Systematic reviews and meta-analyses were synthesized at the level of their published findings, whereas primary studies were considered independently only when directly included in the narrative overview, thereby minimizing duplicate interpretation of the same evidence.

### 2.4 Studies quality

Although a formal risk-of-bias assessment or evidence grading system (e.g., GRADE or Oxford Levels of Evidence) was not applied, consistent with the narrative design of this overview, priority was given to high-quality evidence, including systematic reviews, meta-analyses, randomized controlled trials, and well-designed observational studies. Methodological robustness, study design, relevance to the review objectives, and contribution to the conceptual understanding of the topic were considered during the critical interpretation of the literature. Where appropriate, limitations of individual studies are discussed within the text.

### 2.5 Data synthesis and interpretation

Following study selection, the multidisciplinary research team critically evaluated the relevance of the included studies and extracted the information necessary to support the thematic synthesis of the evidence. Additional relevant references identified through reference screening were incorporated where appropriate to strengthen the conceptual framework.

Due to heterogeneity in study design, populations, and outcomes, a quantitative synthesis was not feasible. Therefore, findings were narratively synthesized and grouped into thematic domains (biological, psychosocial, environmental, and rehabilitative/modulating factors and interventions), allowing the identification of patterns, consistencies, and knowledge gaps. The included studies were organized into thematic domains, and evidence regarding risk and protective factors for depression in children and adolescents with ASD was synthesized and interpreted within a multidisciplinary framework. To facilitate interpretation of the findings, the type of supporting evidence (e.g., systematic reviews, meta-analyses, randomized controlled trials, non-randomized studies, and observational studies) was reported where appropriate; however, no formal grading or hierarchy of evidence was applied, consistent with the objectives of this structured narrative overview. A clear distinction was made between risk and protective factors while also considering their interactions. This integrative approach provided a broader conceptual understanding of the available evidence and highlighted the importance of protective factors alongside risk factors. As previous research has predominantly focused on risk factors for depression in ASD, this narrative overview also emphasizes resilience-oriented and modifiable factors that may inform prevention and intervention strategies.

Any limitations and/or discrepancies identified across the included studies were discussed among the multidisciplinary team to ensure transparency and methodological rigor. The included studies were subsequently considered for thematic and qualitative synthesis, enabling the integration of evidence on both risk and protective factors. This methodological approach was intended to provide a comprehensive synthesis of the available evidence, clarify the interactions between vulnerability and resilience factors, and support the development of a clinically relevant, multidisciplinary conceptual framework for contemporary practice.

## 3 Results

### 3.1 Risk factors associated with depression in ASD children and adolescents

**Figure 1:**
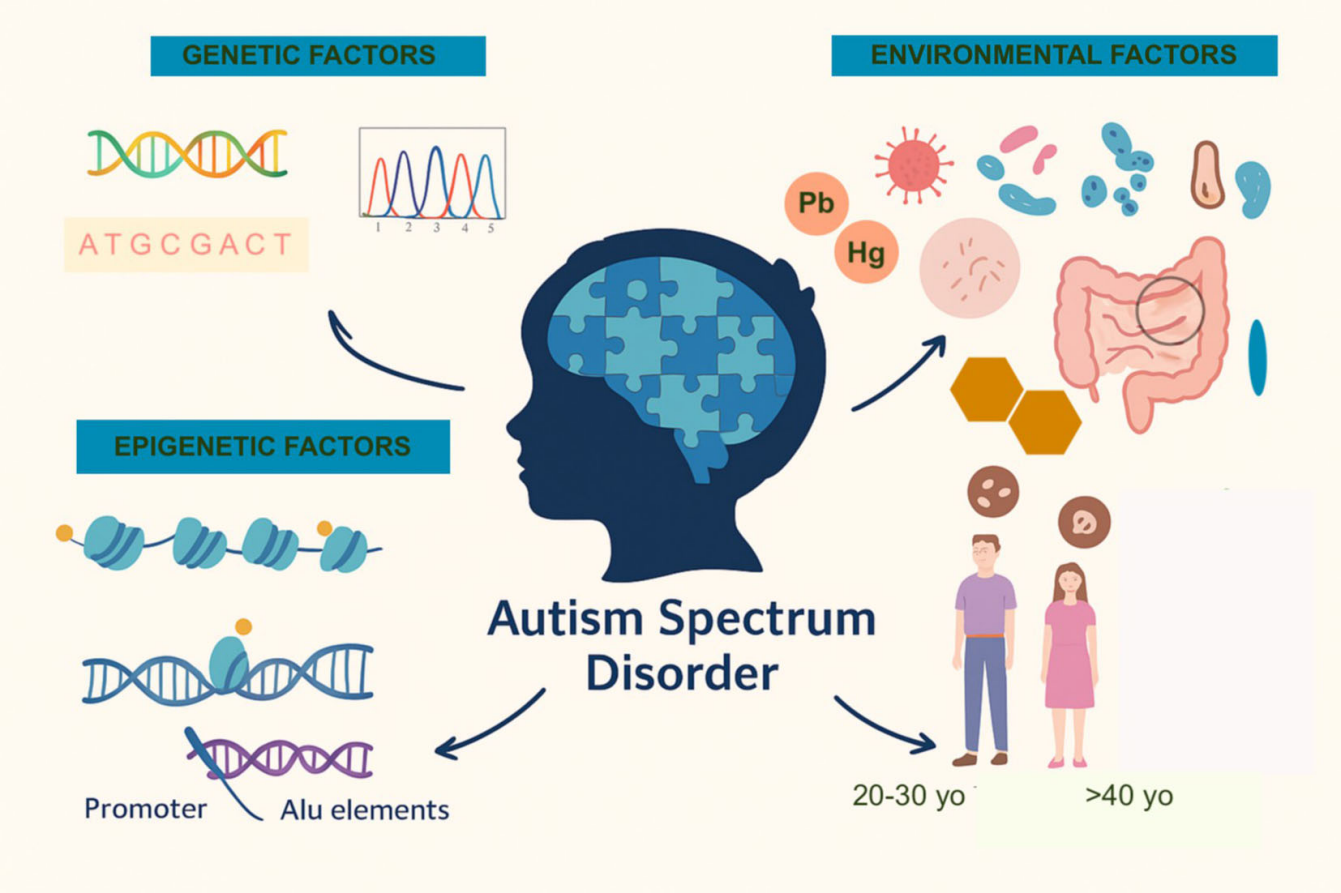
Conceptual overview of the principal genetic, epigenetic, and environmental factors influencing Autism Spectrum Disorder (ASD) in children. The content was developed inductively from the evidence synthesized in the present narrative overview and adapted from Masini et al. (1), International Journal of Molecular Sciences, 21, 8290 (CC BY 4.0). All graphical elements, layout, and visual design were newly created by the authors.

Emerging research has issued several potential factors for depression in autism spectrum disorder (ASD). A recent review of risk factors noted that relatively few longitudinal studies have investigated the relationship between ASD symptoms and the subsequent development of anxiety and depression over time (3). Therefore, summarizing potential risk factors associated with depression in ASD individuals is highly needed to provide an overview which could further broaden the understanding of how these co-factors interact and mark the course of ASD symptoms. The conceptual figures presented in the Results section were developed inductively following the thematic synthesis of the included evidence and were designed to visually summarize the principal risk and protective factors identified during the narrative overview. **Figure 1** summarizes the risk factors linked to ASD onset in children.

For a better standardization, ongoing research has focused on various potentially vulnerable factors for depression in ASD individuals. However, most of the data come from independent studies of ASD and of typically depressed samples, holding no direct comparison of the two populations through time. Some studies have also used longitudinal designs to be able to find the connection between depressed mood and ASD symptoms in time (3).

A meta-analysis comprising sixty-six articles concluded that when compared to typically developing individuals, those diagnosed with ASD are four times more likely to experience depression during their lifetime (13). Data from the meta-analysis indicated that the pooled lifetime and current prevalence was 14.4% and 12.3%. The studies which used a standardized interview to assess depressive disorders (lifetime = 28.5%, current = 15.3%, 95%) and required participants to report their own depressive symptoms (lifetime = 48.6%; current = 25.9%), scored the highest rates of depression. Additionally, depression rates were higher in studies which included participants with higher degree of intelligence. Age and white race were also factors for increasing the risk of depression (40.2% in adult samples vs 7.7% in samples < 18 years old). The evidence suggest that persons with ASD should be screened for depression on a regular basis and offered treatment accordingly (13). Considering current and emerging evidence, better structured interviews for depression assessment should also be considered.

Prevalence trends for individual mental disorders were analyzed across European youth populations and the findings indicated that nearly one in five young people in Europe are affected by a mental disorder, with an overall pooled prevalence of 15.5% (14). Among these, anxiety disorders were the most common, showing a pooled prevalence of 7.9%. This was followed by attention-deficit/hyperactivity disorder (ADHD) at 2.9%, oppositional defiant disorder (ODD) at 1.9%, depressive disorders at 1.7%, conduct disorder (CD) at 1.5%, and autism spectrum disorder (ASD) at 1.4% (14).

An overview reported that based on the parent-rated Pediatric Behavior Scale (PBS) depressive symptoms had higher rates (54%) among youth with ASD and IQ *≥* 80 in comparison to young individuals with ASD and IQ <80 (15). Other findings presented in the overview additionally showed that young individuals with ASD and depression had lower rates of suicidal ideation and suicide attempts in comparison to typically developing young individuals with depression (15). Despite the fact that depression risk rises with age, data show that children with ASD are still at risk of developing symptoms even at a small age (15). Among individuals with ASD, a slight increase in the severity of depression and anxiety was observed from adolescence through middle adulthood, followed by a modest decline in older adulthood (16).

For clarity, the findings are presented according to three overarching domains identified through the thematic synthesis: (i) biological and psychosocial factors, (ii) environmental and lifestyle factors, and (iii) therapeutic and modulating interventions. Within these domains, both risk and protective factors are synthesized according to the available evidence. Because depression in children and adolescents with Autism Spectrum Disorder (ASD) arises from complex interactions among biological, psychological, environmental, and clinical factors, several themes overlap across domains and may encompass both vulnerability and resilience pathways. Therefore, these domains are intended to provide a conceptual framework for organizing the evidence rather than representing mutually exclusive categories.

### 3.2 Biological and psychosocial factors

#### 3.2.1 Implications and interplay of genetic and neurobiological factors

Elevated rates of affective disorders have been observed among family members of individuals with autism, even prior to the birth of a child with a developmental disability (3, 17). Youth with ASD who present with comorbid depression are more likely to have a family history of depression compared to those with ASD without comorbid depression (18). Additionally, not only ASD children and adolescents are affected by depression but also depression rates are higher in first degree relatives of those suffering from ASD in comparison with first degree relatives of those with Down syndrome, when controlling for stress linked to raising a child with ASD (19).

Emerging evidence suggest that ASD, major depressive disorder, and other psychiatric conditions share overlapping genetic risk factors (3, 20). In particular, variants in serotonin and dopamine-related genes have been associated with more severe depressive symptoms in children with ASD (3, 21). Individuals with ASD may be at augmented risk to develop clinically diagnosed depression, as well as anxiety, and bipolar disorder in comparison with age- and sex-matched individuals (22). It is estimated that 32% of the entire relation between social communication impairment and self-harm was explained by symptoms of depression at 12 years (23).

Neurobiological findings further support shared mechanisms between ASD and depression. Abnormalities in serotonin functioning, neuroinflammatory markers such as microglial activation, atypical amygdala structure and function, as well as disruptions in brain connectivity patterns, have all been independently linked to both conditions (3, 24–27).

The findings indicate that subjective perceptions of social impairment and self- awareness of autistic traits contribute significantly to the development of co-occurring depression in ASD, beyond observable symptomatology, and the anterior cingulate cortex (ACC) appears to play a central role in this process. These results suggest that autistic individuals with depression may exhibit distinct biological and perceptual profiles, underscoring heterogeneity within the ASD population (28).

#### 3.2.2 Anxiety, insomnia/sleep problems, intolerance of uncertainty, and sensory sensitivity

Anxiety, insomnia, intolerance of uncertainty (IU), and sensory sensitivity were all factors linked directly with depression, whereas autonomic arousal and sensory sensitivity were additionally correlated indirectly with depression via insomnia (29). Also, autistic traits were correlated indirectly with depression via anxiety (29). Evidence showed that a significant link lays between sensory processing features (SPF) and de- pressive symptoms in boys with ASD (30). One research study found that specific sensory processing patterns, as measured by the Sensory Profile 2 (SP2), are significantly correlated with different emotional and behavioral difficulties, as assessed by the Child Behavior Checklist (CBCL) (31). Scores on CBCL for “anxious/depressed” were positively correlated with low registration and sensory avoiding on the SP2, and “withdrawal/depressed” scores were correlated with sensory avoiding (31).

Neckelmann et al. reported that chronic insomnia may represent a trait marker of depression. However, the available evidence did not support chronic insomnia as an independent state-related risk factor for the subsequent development of depression or indicate a dose-dependent increase in depression risk associated with insomnia (32).

Overall, current evidence suggests that anxiety, sleep disturbances, intolerance of uncertainty, and sensory processing difficulties represent interconnected and potentially modifiable factors associated with depressive symptoms in children and adolescents with ASD. Their complex interactions highlight the importance of comprehensive assessment and early multidisciplinary interventions targeting emotional regulation, sleep quality, and sensory functioning to help reduce vulnerability to depression and improve overall mental health outcomes.

#### 3.2.3 Coping strategies, emotional functioning, loneliness, social communication, bullying and cyberbullying

Depression is often associated with emotional functioning, such as coping strategies, but in children with ASD, difficulties in social interaction also play a significant role (33). In this group, fewer approach-oriented and avoidant strategies, greater reliance on maladaptive coping, and poorer social functioning were linked to higher depressive symptoms. In contrast, among typically developing (TD) boys, only reduced use of approach strategies and greater use of maladaptive coping were related to depression severity (33). Interestingly, boys with ASD who reported using avoidant coping strategies reported fewer depressive symptoms, suggesting that avoidance may serve as an adaptive emotion regulation strategy in this context. However, the influence of over-arousal, the long-term effects of avoidance, and the causal direction of these associations require further investigation (33).

By age 10, children with ASD or elevated autistic traits exhibit higher levels of depressive symptoms compared to the general population, and these symptoms often persist into late adolescence. This risk is especially pronounced in the context of bullying. Among autistic traits, social communication difficulties show a particularly strong link to depression (34). Adolescents with high-functioning ASD who experience bullying victimization exhibit greater severity of depression and anxiety compared to their non-victimized peers (35). ASD high-functioning adolescents often experience adjustment difficulties, notably loneliness and depression (36). Findings indicate that adolescents with ADHD who scored higher on the behavioral inhibition system (BIS), had comorbid ASD, and experienced bullying victimization reported greater severity of both anxiety and depressive symptoms (37). Additionally, adolescents with ADHD who engaged in bullying behaviors exhibited more severe depressive symptoms compared to non-bullying peers. Overall, BIS traits, comorbid ASD, and bullying involvement were significantly associated with anxiety and depression in this population (37). Older age and greater severity of oppositional defiant disorder (ODD) symptoms were significantly linked to both victimization and perpetration of cyberbullying in ASD young individuals. Victimization alone, however, was specifically associated with higher levels of depression, anxiety, and suicidality (38). These findings underscore the need and implications for routine screening of mental health problems in ASD adolescents with a history of bullying (35). Additionally, as an environmental factor, bullying may represent a key target for preventive and therapeutic interventions (34).

Regarding loneliness and social interaction, it was observed that children and youth with ASD experience significantly higher levels of loneliness than their neurotypical peers, and this loneliness is moderately to strongly associated with depression and anxiety (39).

Overall, current evidence indicates that emotional functioning, maladaptive coping strategies, social communication difficulties, loneliness, and experiences of bullying or cyberbullying represent closely interconnected psychosocial factors contributing to depressive symptoms in children and adolescents with ASD. These findings emphasize the importance of early identification of psychosocial vulnerabilities and the implementation of individualized interventions that strengthen adaptive coping skills, promote social inclusion, and prevent bullying, thereby reducing the risk of depression and improving emotional well-being.

### 3.3 Intelligence quotient and verbal IQ

A non-randomized trial including a total of 1,272 participants aged 6 to 17 years (mean [SD] age = 9.56 [2.79] years) from the Autism Speaks Autism Treatment Network, stated that 7.0% of children with ASD had a reported history of depression, including 4.8% of those aged 6–12 years and 20.2% of those aged 13–17 years. A history of depression was significantly associated with older age, higher IQ, and a diagnosis of Asperger’s disorder (40). After adjusting for age, IQ, and ASD subtype, the ever-depressed group showed significantly higher rates of seizure disorders (odds ratio = 2.64) and gastrointestinal issues (odds ratio = 2.59). There were also trend-level differences in aggression, somatic complaints, and social difficulties. No significant group differences were found in autism symptom severity, repetitive behaviors, sleep or eating disturbances, self-injurious behavior, or current treatment use (40).

A survey including 165 participants aged between 6 and 24 years found that higher verbal IQ (VIQ) was associated with increased anxiety, and after controlling for VIQ, both anxiety and depression were more pronounced in individuals with ASD compared to non-ASD peers (41). Female sex was associated with a greater increase in anxiety and depressive symptoms over time in both ASD and non-ASD populations, with a more pronounced rise during adolescence (16). Conversely, males with ASD demonstrated elevated depressive symptoms beginning in childhood that persisted into young adulthood (41).

Taken together, the available evidence suggests that higher cognitive functioning, particularly higher IQ and verbal IQ, may be associated with an increased recognition and reporting of depressive and anxiety symptoms in some individuals with ASD, especially during adolescence. These findings indicate that cognitive ability should be considered a vulnerability stratification factor rather than a direct risk factor, highlighting the need for individualized mental health assessment across different cognitive profiles.

### 3.4 Concurrent physiological factors: age, menarche, puberty

Differences in pubertal timing are considered risk factors for attaining poor out- comes related to mental health, notably early maturation for girls and late maturation for boys, and advanced pubertal timing has been correlated with augmented rates of depression (42). Regression results indicated that age, girls’ social anxiety, and mothers’ depression were major factors to contribute to depression scores, but with different effects. ASD girls’ self-reports of their depression were highly associated with their HPA-axis responses, but not with their status of menarche (43). In autistic adolescents, factors such as social impact, verbal skills, and strong social motivation can increase the risk of developing depression (44). Childhood social impairments are linked to peer victimization, which further elevates this risk. However, currently, there is no assessment tool specifically designed to measure depression in autistic adolescents (44).

A four-year longitudinal study followed 244 participants aged 10–13 years, including 140 youths with ASD (36 females) and 104 typically developing (TD) peers (46 females), using linear and logistic mixed-effects models (42). Among participants with ASD, depressive symptoms were elevated during early adolescence but gradually decreased throughout middle adolescence and puberty. In contrast, the TD group showed the opposite pattern, with depressive symptoms increasing as development progressed. In the ASD group there were augmented depressive scores in early adolescence and they decreased during middle adolescence and puberty, whereas in the TD group it was described an opposite trend showing a rise in symptoms of depression in line with advancing development. However, these differences in pubertal timing are considered risk factors for poor outcomes related to mental health, notably early maturation for girls and late maturation for boys. Advanced pubertal timing has been linked to increased rates of depression (42). Youngsters suffering from concurrent ASD/gender dysphoria (GD) were at greater risk of developing depression in comparison with youth with ASD alone, GD alone, or none of the two diagnoses (45).

### 3.5 Environmental and lifestyle factors

#### 3.5.1 Co-occuring health issues and mental health conditions and overrepresentation of ASD diagnosis

Research indicates that ASD diagnoses and autistic traits are overrepresented among gender diverse populations, especially in children and adolescents (46). Current evidence suggest that gender diverse youth with ASD represent a particularly vulnerable group, facing elevated risks for mental health challenges—especially internalizing disorders—and reduced quality of life (46). Services supporting gender diverse young people should therefore include routine ASD screening and ensure access to appropriate care for co-occurring mental health difficulties (46). Co-occurring conditions may further influence the risk of depression in individuals with ASD. For example, some studies have reported high rates of depression, anxiety, and particularly social phobia among deaf and hard-of-hearing children and adolescents. However, other evidence suggests that the overall prevalence of affective disorders in hearing-impaired youth is comparable to that reported in their normal-hearing peers (47). Evidence showed that a significant percentage of children with ASD included in the study presented at least one additional psychiatric disorder (4). Besides social anxiety disorder, attention-deficit/hyperactivity disorder, and oppositional defiant disorder, the study of Bailly also identified Diagnostic and Statistical Manual of Mental Disorders (DSM)-IV diagnoses for depressive and conduct disorders, as well as tic disorders, enuresis, encopresis, and trichotillomania (4).

Overall, current evidence suggests that co-occurring medical, neurodevelopmental, and psychiatric conditions substantially contribute to the complexity of depression in children and adolescents with ASD. Rather than representing isolated comorbidities, these conditions may interact with ASD-related vulnerabilities to influence mental health trajectories. These findings highlight the importance of comprehensive multidisciplinary assessment, routine screening for co-occurring disorders, and individualized care pathways to facilitate early identification and timely intervention.

#### 3.5.2 External factors: restrictions during pandemic, negative life events and maltreatment

A recent systematic review involving data from over 55,000 children and adolescents worldwide (mean age 11.3 years) found high rates of emotional and behavioral difficulties for anxiety, depression, irritability, and anger (48). Nevertheless, the impact of the pandemic’s was not uniformly spread across all youth. Risk factors for poorer mental health included pre-existing mental health problems, excessive exposure to media, and also living in areas with increased COVID-19 cases. However, maintaining family routines, as well as effective and positive parent–child communication were found to be protective factors (48).

When compared to matched controls, youngsters with ASD have reported augmented negative life events one year prior to the onset of depression (49).

Regarding life experiences, parental reports of their children’s experiences of depression were largely consistent with the children’s self-reported perspectives. Novel contributions included descriptions of depression-associated dietary restriction and the masking of mental health difficulties. Both parents and children emphasized a perceived link between autism and the onset of depression, underscoring the challenges of navigating a complex, predominantly neurotypical social environment (50).

Maltreatment and autism can be linked with overlapping difficulties on multiple functional domains, including social, emotional, and sensory domain and high rates of mental health issues (51). Evidence indicated that a cognitive approach focusing on affect-laden cognition, as future thinking (FT), could enhance informed cognitive assessments and also adapt psychological interventions for these individuals. When comparing three different groups (autism, maltreatment, and matched peers typically developing without maltreatment/autism from 10 to 16 years old), exploratory analyses indicated that lower FT specificity was highly linked with more depressive symptoms, but not anxiety (51).

Taken together, the available evidence indicates that adverse environmental experiences—including pandemic-related restrictions, negative life events, and maltreatment—may increase vulnerability to depressive symptoms in children and adolescents with ASD. Conversely, supportive family environments, stable daily routines, and positive parent–child relationships appear to mitigate these adverse effects. These findings reinforce the importance of adopting trauma-informed, family-centered, and resilience-oriented approaches when assessing and managing depression in young people with ASD.

To facilitate interpretation of the findings, the principal risk factors influencing depression in children and adolescents with ASD are summarized in **Table 1** according to their proposed mechanisms, target domains, and supporting evidence.

**Table 1:** Principal risk factors influencing depression in children and adolescents with autism spectrum disorder (ASD).

Risk factor	Description/mechanism	Target domain	Supporting evidence
Genetic and neurobiological vulnerability	Family history, neurodevelopmental alterations, sensory dysregulation, autonomic dysfunction, intolerance of uncertainty, sleep disturbances, emotional dysregulation	Increased susceptibility to depressive symptoms and impaired emotional regulation	Systematic reviews/Meta-analyses/Longitudinal studies (3, 17-32)
Maladaptive coping, emotional dysregulation, loneliness, impaired social communication, bullying and cyberbullying	Reduced adaptive coping strategies, social isolation, peer victimization, communication difficulties, loneliness, cyberbullying	Increased depressive symptoms, anxiety, social withdrawal, suicidality	Systematic reviews/Meta-analyses/Cohort studies/Cross-sectional studies (33–39)
Higher IQ and verbal IQ (VIQ)	Greater awareness of social differences, camouflaging, increased insight into interpersonal difficulties	Higher risk of internalizing symptoms, particularly depression during adolescence	Longitudinal studies/Non-randomized studies/Surveys (16,40,41)
Co-occurring psychiatric and medical conditions	ADHD, anxiety disorders, seizure disorders, gastrointestinal disorders, hearing impairment, other psychiatric comorbidities	Increased clinical complexity and vulnerability to depression	Clinical studies/Observational studies/Systematic reviews (4,46,47)
Environmental stressors and adverse life experiences	COVID-19 restrictions, negative life events, maltreatment, chronic stress, adverse family or social experiences	Increased emotional distress and depressive symptoms	Overview/Cohort studies/Qualitative studies/Semi-structured interviews (48–51)

## 4 Protective factors for depression in ASD

**Figure 2:**
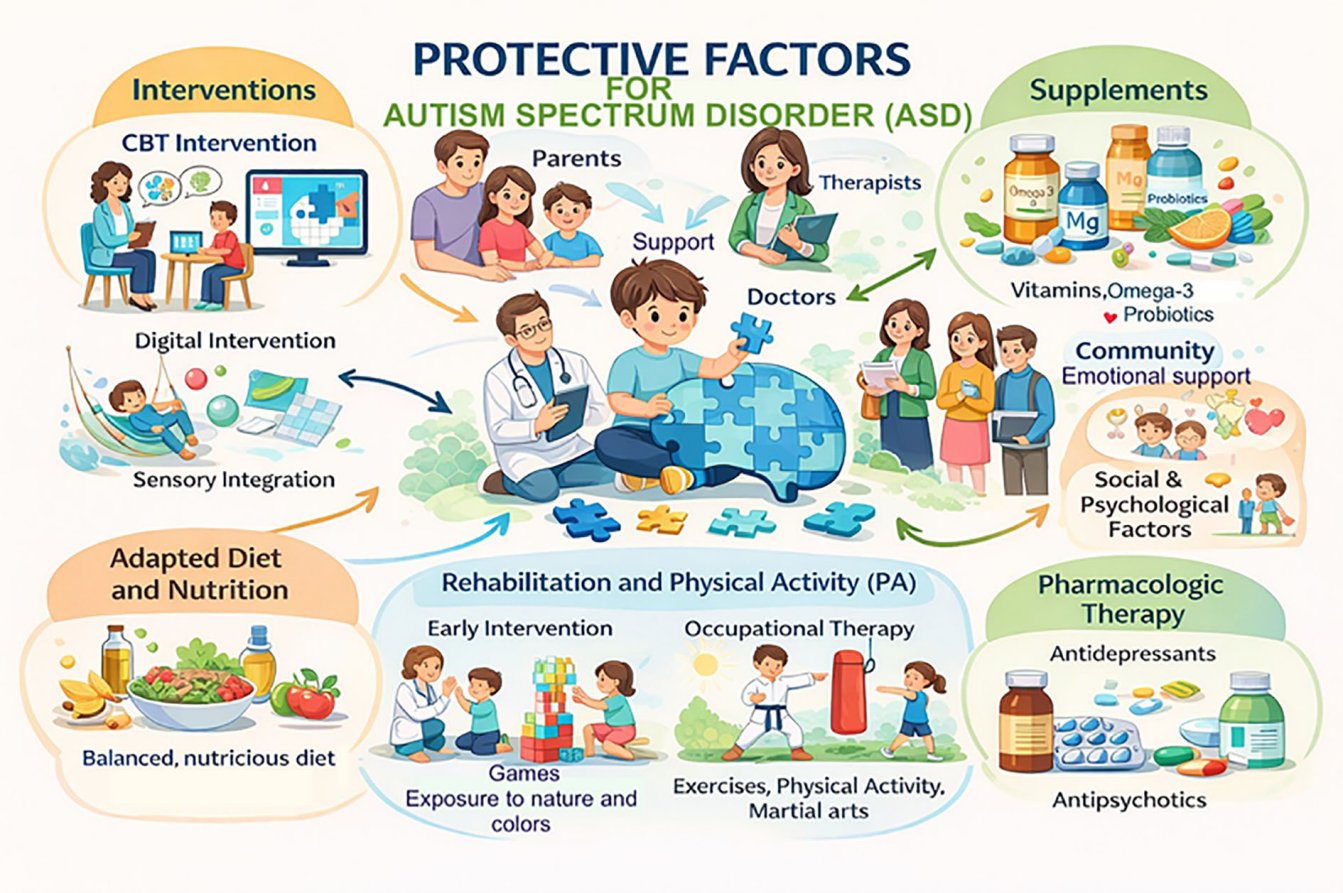
Conceptual overview of protective factors influencing depression in children with autism spectrum disorder. Original schematic conceptually designed by the authors to illustrate and summarize protective factors influencing mental health outcomes in children with ASD. The visual representation was initially generated using an AI-assisted tool and subsequently manually refined and edited by the authors using standard software tools. The figure was developed inductively from the evidence synthesized in the present narrative overview (references 9,10,54–90) to provide a visual summary of the principal protective factors and potential intervention targets. CBT, cognitive behavioral therapy; PA, physical activity.

**Figure 2** outlines a conceptual overview on the role and interplay of various factors interfering with depression in children diagnosed with ASD.

### 4.1 Environmental and lifestyle factors

#### 4.1.1 Routines

The presence of family routines and effective parent-child communication were protective factors for youth with ASD during COVID-19 pandemic (48). Adolescents with ASD are often experiencing poor socio-emotional adjustments and interactions with their peers, but having a pet and taking care of it could serve as an intermediary. Findings indicate that pet ownership may promote improved social-emotional adjustment in adolescents with ASD (52). Even though evidence may show a beneficial effect of adhering to enjoyable routines, each individual has its own way of implementing them, thus drawing a clear path and assess the best ones requires more in depth research studies.

Overall, the available evidence suggests that maintaining predictable daily routines and fostering positive family interactions may represent accessible protective strategies for reducing depressive symptoms and promoting emotional well-being in children and adolescents with ASD. Nevertheless, the optimal type, frequency, and individualization of these routines remain insufficiently defined, highlighting the need for further longitudinal and intervention studies.

#### 4.1.2 Vitamins, minerals, Omega-3 and other supplements

Broad-spectrum formulas (vitamins and minerals) demonstrated more robust effects than formulas with fewer ingredients and indicated a pattern of beneficial effects in autism, and in participants with behavioral deficits in dementia (53). A three month controlled 20 mg thiamine vitamin/mineral treatment study showed significant improvements in the metabolic status of children with ASD. The degree of improvement on the average change of the Parental Global Impressions-Revised (PGI-R) was highly linked to several biomarkers with baseline levels of biotin and vitamin K being the most significant, both of them being made by the beneficial intestinal flora (54). Additionally, vitamin supplementation has been shown to improve or normalize levels of glutathione, plasma adenosine triphosphate (ATP), nicotinamide adenine dinucleotide (NADH), nicotinamide adenine dinucleotide phosphate (NADPH), plasma sulfate, and biotin, while simultaneously reducing oxidative stress biomarkers in children with ASD (55). Serum retinol and 25-hydroxy vitamin D levels were found to be significantly lower in children with autism compared to controls. Moreover, combined deficiencies of vitamin A and vitamin D appear to have a greater impact on symptom severity and developmental outcomes in autistic children (56). Individuals with mild to moderate depression who received omega-3 fatty acid supplementation and a single antidepressant showed significantly greater improvements in depressive symptoms compared to receiving only omega-3 fatty acid supplementation or a single antidepressant (57). Vitamin B12 intake was also linked with decreased risk of depression (58). Evidence also indicated improvement of core ASD symptoms by using omega-3-fatty-acids, carnosine, haloperidol, folinic acid, guanfacine, probiotics, sulforaphane, tideglusib, and valproate (59). Evidence showed that carnosine supplementation (800 mg/day) for 10 weeks led to a decrease in hyperactivity and non-compliance in children with ASD (60). Carnosine, taurine, and glutathione have been investigated for their potential neuroprotective effects, with taurine showing particular promise in enhancing cognitive function and reducing neurotoxicity (61). Probiotic supplementation—most commonly involving mixtures of Bifidobacteria, Streptococci, and Lactobacilli—appears to be one of the most promising approaches for addressing both neurobehavioral symptoms and gastrointestinal dysfunction in individuals with ASD, although existing clinical trials remain limited and heterogeneous (61, 62). Dietary interventions, such as gluten- and casein-free diets, have been associated with reduced digestive issues, improved mealtime behaviors, enhanced eye contact and communication, and greater social engagement. The ketogenic diet has demonstrated neuroprotective properties while also helping to reduce food selectivity and neophobia (61, 63). Supplementation with fatty acids has similarly been linked to improvements in ASD-related symptoms. Furthermore, after 26 weeks of treatment, phenolic compound formulations were shown to reduce autistic behaviors and significantly lower serum levels of TNF-*α* and IL-6, likely due to their anti-inflammatory effects (61). A magnesium–vitamin B6 (Mg–B6) regimen (6 mg/kg/day Mg, 0.6 mg/kg/day vitamin B6) administered for at least 8 weeks was associated with reductions in hyperactivity and hyperemotivity/aggressiveness, alongside improvements in school attention (64). This treatment also resulted in a significant increase in erythrocyte magnesium (Erc-Mg) levels. However, upon discontinuation of the Mg–B6 regimen, clinical symptoms re-emerged within a few weeks, accompanied by a decline in Erc-Mg values. Social interactions, restricted behavior, communication, and delayed functioning were also improved, and clinical symptoms were scored 0 to 4 (64). Additionally, natural vitamin A may reconnect the retinoid receptors which are of paramount importance for vision, sensory perception, language processing, and attention (65). Overall, given the heterogeneity and variability in approaches, more robust studies are needed to be able to draw a conclusion on the efficiency of the supplements usage and data should be considered with attention.

Taken together, nutritional interventions and dietary supplementation represent promising complementary approaches for supporting mental health and behavioral functioning in ASD. However, the current evidence remains heterogeneous with respect to formulations, dosages, study designs, and clinical outcomes. Consequently, these interventions should be considered adjunctive rather than established treatments for depression, and larger well-designed randomized controlled trials are needed to clarify their role.

#### 4.1.3 Colors

Colors play an important role for the spaces of children with mental health disorders (MHD). It was noted that blue and green spaces can decrease emotional, behavioral, and social issues in disabled children. Evidence showed a protective correlation between the level of greenness or blue and depressive and anxiety symptoms. Moreover, even after adjusting for socioeconomic confounders, in most of the studies there were no significant changes in the results (66). There is growing evidence for the environmental microbiota–human health pathway, alongside moderate to strong support for broader nature-based pathways—such as exposure to green spaces—being linked to multiple health benefits, including stress reduction, improved well-being, and enhanced social connectedness (67). Mavoa et al. investigated the impact of neighborhood green spaces and biodiversity on the emotional well-being of 4,575 adolescents in New Zealand. The study found a significant association between lower levels of depressive symptoms and greater exposure to green spaces, presence of native vegetation, and higher scores on the nature availability index (68).

Overall, exposure to natural environments and green or blue spaces appears to be associated with improved emotional well-being and lower depressive symptom burden in young individuals, including those with ASD. Although these findings support environmental modification as a potentially valuable component of holistic mental health care, additional ASD-specific longitudinal and interventional studies are required to establish causality and determine optimal implementation strategies.

### 4.2 Games and digital health interventions

New technologies and video games can help overcome traditional barriers to care by enabling evidence-based therapy through teleconferencing while simultaneously offering engaging, interactive experiences (69). Overall, video game–based interventions have demonstrated usefulness and effectiveness in reducing symptoms of depressive disorders (69). Additionally, it was noted a growing research interest regarding this therapeutic approach. When adherence and feasibility were assessed, both acceptability and engagement levels were consistently high (69). Digital health interventions (DHIs) show promising clinical benefits, particularly computerized cognitive behavioral therapy (cCBT), for treating depression and anxiety in adolescents and young adults (70). A recent systematic review identified 30 new randomized controlled trials evaluating DHIs across conditions including ADHD, autism, anxiety, depression, psychosis, eating disorders, and post-traumatic stress disorder (PTSD) (70). While evidence may support the effectiveness of cCBT for internalizing disorders, the benefits of DHIs for ADHD, autism, psychosis, and eating disorders remain uncertain. Overall, current evidence suggests that digital health interventions, particularly cCBT and game-based approaches, represent promising complementary strategies for improving depressive symptoms and treatment engagement in young people with ASD. Nevertheless, the evidence remains heterogeneous, and further ASD-specific randomized controlled trials with long-term follow-up are needed to establish their effectiveness and cost-effectiveness (70).

### 4.3 Natural environment

Beyond diet, environmental factors such as exposure to nature may also support mental health in children and adolescents. Lomax emphasized the beneficial effects of natural environments on well-being, although evidence were mixed for certain outcomes, including self-concept, problem-solving, mood, emotional well-being, self-esteem, and depression (71). Among two cohort studies reviewed, one confirmed nature’s positive influence on improving mental well-being, reinforcing the need to integrate environmental and lifestyle interventions alongside dietary strategies for holistic support in pediatric populations (71). Taken together, exposure to natural environments appears to represent a potentially beneficial lifestyle-based strategy for supporting emotional well-being in children and adolescents with ASD. However, current evidence regarding its effects on depressive symptoms remains limited and inconsistent, warranting further well-designed longitudinal and intervention studies before firm clinical recommendations can be made.

## 5 Interventions for modulating depression in children with ASD

### 5.1 Therapeutic and modulating interventions

#### 5.1.1 Cognitive behavioral therapy

A comprehensive umbrella review encompassing 93 systematic reviews (including 393 randomized controlled trials) reported that behavioral interventions incorporating CBT improved sleep parameters and associated mood disturbances in children with insomnia, ADHD, and ASD, although the overall certainty of the evidence was considered low (72). Overall, the available evidence consistently identifies CBT as one of the best-supported psychosocial interventions for managing depression and co-occurring anxiety in children and adolescents with ASD (72–74). When appropriately adapted to the individual’s cognitive, communication, and sensory profile, CBT may improve emotional regulation, coping strategies, stress management, and depressive symptoms (74). Although treatment protocols and outcome measures remain heterogeneous across studies, current evidence supports CBT as a cornerstone of multidisciplinary mental health care for young people with ASD, particularly when integrated with family involvement and other individualized psychosocial interventions.

#### 5.1.2 Physical activity and structured interventions

Physical activity (PA) and structured exercise interventions have shown significant benefits for individuals with ASD, influencing behavioral, cognitive, social, and emotional outcomes. Toscano et al. demonstrated that a 48-week exercise program consisting of 30-minute sessions twice per week decreased social interaction problems, attention deficits, emotional reactivity, stereotypic behaviors, and sleep disturbances in children with ASD, although eye contact and food selectivity remained unaffected (75). Even though these outcomes are not direct indicators of depression, they represent clinically relevant factors that have been associated with emotional functioning and vulnerability to depressive symptoms in individuals with ASD.

Beyond behavioral outcomes, physical activity also enhances cognitive function and executive processes in ASD populations (75, 76). A systematic review and meta-analysis reported that exercise interventions produced large improvements in working memory and response inhibition in children with ASD, though methodological heterogeneity was noted (76). Liang et al. confirmed these findings, showing small to moderate effects of chronic exercise on cognitive flexibility and inhibitory control, though not on working memory (77). Bremer et al. further highlighted that activities such as jogging, horseback riding, martial arts, swimming, yoga, and dance led to meaningful gains in stereotypic behaviors, cognition, attention, and socio-emotional functioning, with horseback riding and martial arts producing the largest effect sizes (78).

A systematic review reported that aquatic interventions delivered by professionals trained in both behavioral support and aquatic therapy significantly enhanced motor abilities and social engagement while reducing autistic behaviors in children with ASD (79). Their results highlight the value of structured, multidisciplinary aquatic programs as part of ASD rehabilitation (79). A physical activity–based intervention was implemented in twelve participants aged 15–27 years, who attended 90-minute sessions twice weekly for six weeks and were encouraged to increase daily activity levels (80). Depressive symptoms were assessed using the Patient Health Questionnaire-9 (PHQ-9) at baseline and post-intervention. Results demonstrated a significant reduction in depression severity, with mean PHQ-9 scores decreasing from mild to minimal levels by program completion (80).

Overall, the evidence demonstrates that PA offers broad and meaningful benefits for individuals with ASD, spanning cognitive, motor, social, and emotional domains. While exercise does not uniformly improve all autism-related challenges, it consistently supports better mental health, executive functioning, and quality of life outcomes. The findings highlight the importance of individualized, sustained, and diverse PA interventions—ranging from aerobic exercise and martial arts to water-based activities and structured sports—in addressing the complex needs of this population (10, 80).

PA plays a crucial role for emotional and psychological well-being in individuals with ASD. One study found a negative association between physical activity and anxiety/depression across youth populations, including those with ASD, though high rates of comorbid anxiety and depression persisted (81). Wiggins et al. demonstrated that participation in sports was associated with a lower likelihood of depressive symptoms, yet autistic adolescents were less engaged in physical activity compared to peers with other developmental disorders or neurotypical controls (82). Shahane emphasized the importance of PA in underserved populations such as young adults with ASD, noting consistent improvements in physical fitness, motor skills, psychological function, and QoL (83).

Social impairment emerged as a key mediating factor linking all examined execu- tive function (EF) domains to overall adjustment, whereas friendship quality partially mediated the association between emotional control and loneliness. These findings underscore the importance of interventions that target both social skills and broader adjustment in adolescents with ASD (36).

Evidence suggests that several practical strategies may improve sleep and emotional well-being in children and adolescents with ASD. These include the use of individualized sensory and relaxation tools before and during bedtime, participation in regular daytime physical activities, and allocating time for preferred hobbies or activities of personal interest. Establishing predictable and enjoyable family routines before bedtime has also been associated with positive outcomes (84). Additionally, spending time with family members in predictable and enjoyable routines prior to bedtime was also emphasized (84).

Taken together, the available evidence supports structured physical activity as one of the most consistently beneficial non-pharmacological interventions for children and adolescents with ASD. Although improvements in depressive symptoms are often indirect—through enhanced emotional regulation, sleep quality, executive functioning, social participation, and overall well-being—exercise-based interventions represent practical, low-risk, and clinically meaningful strategies that can be incorporated into multidisciplinary care. Future studies should further determine the optimal type, intensity, frequency, and duration of exercise for maximizing mental health outcomes in this population.

The principal protective factors and therapeutic strategies identified in the literature are summarized in **Table 2** according to their proposed mechanisms, target domains, and supporting evidence.

**Table 2:** Principal protective factors and therapeutic interventions influencing depression in children and adolescents with autism spectrum disorder (ASD).

Protective factor	Description/mechanism	Target domain	Supporting evidence
Vitamin & Nutritional Supplementation (e.g., magnesium, omega-3 fatty acids); Dietary Interventions (balanced, anti-inflammatory, microbiome-supportive diets)	Supports neurodevelopment, synaptic function, inflammation regulation, and mood stabilization	Emotional regulation, cognition, behavior	Systematic reviews/Meta-analyses/Clinical trials/Pilot studies/ Case-control study (54–65)
Digital Health Interventions (apps, serious games, tele-intervention)	Improve accessibility, engagement, monitoring, and continuity of care	Social skills, anxiety, emotional regulation	Systematic reviews/Meta-analyses (69,70)
Cognitive Behavioral Therapy (CBT)	Targets anxiety, emotional regulation, and maladaptive thought patterns	Anxiety, depression, coping skills	Systematic reviews/Meta-analyses (74–76)
Sensory Integration Therapy (e.g., weighted blankets, sensory integration, and environmental modulation)	Addresses sensory processing differences to improve participation and behavior	Sensory modulation, daily functioning	Systematic reviews/Meta-analyses/Stakeholder-driven study (85–88)
Exercise & Physical Activity (including martial arts)	Enhances motor skills, emotional regulation, self-esteem, and social interaction	Motor skills, mental health, socialization	Systematic reviews/Meta-analyses/Stakeholder-driven study/Clinical trials (9,10,75–84)
Use of Colors (Environmental & Therapeutic)	Can influence sensory comfort, attention, and emotional regulation	Sensory regulation, behavior	Scoping reviews/Systematic review (66–68,71)
Pharmacological Treatment (antipsychotics, antidepressants when indicated)	Reduces severe irritability, aggression, anxiety, or depressive symptoms when clinically necessary	Behavioral regulation, mood	Systematic reviews/Meta-analyses/Observational study (59,61,89,90)

#### 5.1.3 Sensory interventions (e.g. weighted blankets, sensory integration, and environmental modulation)

A meta-analysis of eight studies (n=426) from Zhao et al. found that weighted blankets produced a small but significant reduction in anxiety levels and a potential improvement in insomnia severity (MD=-2.78, p=0.001 in sensitivity analysis). No serious adverse events were reported, though evidence quality was limited by high risk of bias (85). Exposure to neutral sensory environments was found to support emotional regulation and behavioral stability in individuals with heightened sensory sensitivities (86).

Sensory integration interventions have been shown to reduce emotional and behavioral difficulties, including hyperactivity, aggression, anxiety, depression, somatization, attention and learning problems, atypical behaviors, and social withdrawal, in children with ASD (87). Evidence highlighted the potential efficacy of repeated Snoezelen room sessions in reducing the severity of ASD symptoms, particularly repetitive and stereotyped behaviors, as reflected in the Childhood Autism Rating Scale (CARS) scale (88).

Overall, sensory-based interventions appear to represent promising complementary approaches for improving emotional regulation and reducing anxiety, depressive symptoms, behavioral difficulties, and sensory distress in children with ASD. Although the current evidence is encouraging, it remains heterogeneous and is limited by relatively small studies and variable methodological quality. Consequently, these interventions should be considered individualized adjunctive strategies within multidisciplinary rehabilitation programs while further high-quality trials establish their long-term effectiveness.

#### 5.1.4 Pharmacologic therapy

Pharmacological treatment should generally be considered an individualized, adjunctive strategy that complements behavioral, psychosocial, educational, and family-centered interventions, which remain the cornerstone of ASD management and mental health care. Pharmacotherapy primarily targets co-occurring psychiatric symptoms, including depression, anxiety, irritability, sleep disturbances, and behavioral dysregulation, rather than the core features of ASD itself.

Pharmacological therapy, particularly antidepressants, has been investigated for the management of depression, anxiety, and behavioral symptoms in children and adolescents with ASD, although the available evidence remains heterogeneous. Tricyclic antidepressants (TCAs), such as clomipramine and tianeptine, have shown inconsistent short-term benefits on selected ASD-related symptoms, while frequent adverse effects and high dropout rates have limited their clinical applicability (89).

A large meta-review of randomized controlled trials reported that antidepressant efficacy is disorder-specific, with fluoxetine demonstrating benefits for major depressive disorder, fluvoxamine and paroxetine for anxiety disorders, and fluoxetine or sertraline for obsessive-compulsive disorder. Within ASD populations, clomipramine and tianeptine demonstrated modest improvements in selected symptom domains but were associated with poorer tolerability. Overall, the evidence is limited by the predominance of short-term studies and safety concerns, including the increased risk of suicidality reported with paroxetine and venlafaxine in young people (90).

Adjunctive strategies may further enhance treatment outcomes. For example, a randomized trial demonstrated that combining omega-3 fatty acid supplementation with antidepressant therapy resulted in significantly greater improvements in depressive symptoms compared with antidepressant therapy or supplementation alone (57).

Although antidepressants remain the primary pharmacological option for clinically significant depressive symptoms, and adjunctive antipsychotics may be considered in selected cases presenting with severe irritability or behavioral dysregulation, current evidence supports cautious, individualized, and closely monitored prescribing. Pharmacological treatment should therefore be integrated within multimodal rehabilitation programs, alongside psychological therapies, behavioral interventions, educational support, and family-centered care, rather than being used as a standalone treatment (57, 89, 90). Beyond antidepressants, several medications—including atomoxetine, bumetanide, aripiprazole, and risperidone—have demonstrated improvements in specific symptom domains compared with placebo; however, these agents primarily target associated comorbid symptoms rather than depressive symptoms or the core features of ASD (59). Nevertheless, further high-quality ASD-specific studies are needed to establish the long-term efficacy, safety, and optimal use of pharmacological interventions, particularly for depressive symptoms, given the marked heterogeneity in clinical presentation, treatment response, tolerability, and co-occurring medical conditions.

## 6 Discussion

To enhance integration, this narrative overview adopts a biopsychosocial–developmental framework, in which depression in ASD is understood as the result of dynamic interactions between: biological vulnerability (genetic, neurodevelopmental factors), psychological processes (emotion regulation, cognitive style), and social/environmental context (family, stigma, applied interventions) (1–3). Within this model, risk factors increase vulnerability, while protective factors promote resilience and adaptive functioning, offering potential targets for intervention (1–3, 33, 61, 62, 70, 72, 77, 78, 83, 86, 87). Depression represents a highly prevalent yet often underrecognized dimension of ASD, with profound consequences for functional outcomes, QoL, and long-term well-being in this vulnerable population (1, 2, 4–6, 28, 29, 63).

While anxiety frequently co-occurs with depression in ASD and is occasionally referenced for contextual purposes, the primary focus of this overview remains on depressive symptoms and related factors. This contextual consideration is important because anxiety and depression frequently share risk factors, developmental pathways, and functional consequences in ASD, and interventions targeting one domain may indirectly influence the other. This narrative overview highlights that mental health vulnerabilities in children with ASD arise from a complex interplay of biological, environmental, and psychosocial factors, many of which are potentially modifiable (1–4). While existing literature has traditionally emphasized risk pathways, the present synthesis underscores the equal importance of identifying and strengthening protective factors that can buffer emotional distress and foster resilience (1–5, 8, 14). By integrating evidence from high-quality systematic reviews, meta-analyses, and robust clinical studies, this work provides a balanced framework that moves beyond deficit-based models toward a more comprehensive understanding of mental health in ASD.

The synthesis of literature highlights that risk factors for depression in ASD overlap significantly and often interact (1–5, 28, 29, 63). The evidence emphasize how demographic factors like gender and IQ can function as both risk and protective influences depending on context (15, 16, 40, 41). Compared to broader literature, the findings confirm known patterns — such as the higher vulnerability of females due to under-diagnosed camouflaging — and highlight the emerging promise of non-pharmacological interventions (e.g., sensory integration, animal-assisted therapy, diet) (1–5, 16, 41).

A central contribution of this overview is the emphasis on lifestyle-based protective strategies, including physical activity, structured exercise, psychosocial and behavioral therapies, sensory integration, digital health interventions, and family-centered care. These approaches may exert beneficial effects through neurobiological mechanisms, improved emotional regulation, enhanced social participation, and increased self-efficacy. Importantly, such interventions are not merely adjunctive but represent clinically actionable pathways that can be personalized and integrated into multidisciplinary care models. Early diagnosis and timely intervention further amplify these protective effects, highlighting the critical window during childhood for modifying developmental trajectories and improving long-term mental health outcomes. This perspective has practical implications because it shifts clinical attention from the identification of deficits alone toward the active cultivation of resilience, adaptive coping, family support, and environmental accommodations that may reduce vulnerability to depression over time.

Overall, while these interventions show promise, the current evidence base remains heterogeneous, and well-designed ASD-specific randomized controlled trials are still limited. Many reported associations remain correlational and should not be interpreted as causal. Nevertheless, the available evidence supports a shift toward prevention-oriented and personalized models of care that balance the identification of vulnerability with the active promotion of resilience. From a clinical perspective, these findings support routine screening for depressive symptoms within pediatric and child psychiatry services, multidisciplinary assessment of modifiable psychosocial and lifestyle factors, and the early implementation of individualized interventions integrated into rehabilitation programs. At the public health level, the findings emphasize the importance of family-centered care, school-based mental health support, community inclusion, and equitable access to evidence-based interventions to reduce the long-term burden of depression in children and adolescents with ASD.

### 6.1 Limitations

This paper also has some limitations that are addressed further. Whereas this narrative overview highlights multiple modifiable factors, the current literature is characterized by methodological variability, small sample sizes, and a predominance of cross-sectional designs. These limitations restrict causal inference and emphasize the need for longitudinal and intervention-based research. Furthermore, the integration of risk and protective factors remains largely conceptual, underscoring the need for future studies to develop and validate comprehensive models. Importantly, not all interventions are equally supported by evidence, and some (e.g., dietary supplements, environmental modifications) remain exploratory. Given that ASD is a highly heterogeneous condition, variability may be present in cognitive functioning, language ability, age, gender, and comorbidities. These factors may significantly influence both the expression of depressive symptoms and response to interventions. However, most available studies do not stratify findings according to these variables, limiting the ability to draw personalized or subgroup-specific conclusions. Despite these drawbacks, this narrative overview summarized and stratified the most frequent modifiable factors for ASD in children and adolescents, blending the already established risk factors with factors promoting resilience and highlighting that a change in paradigm is also needed: the shift from risk pathways has to be done by using integrative and adaptable interventions to help decrease the burden of MHD and increase QoL in vulnerable populations.

### 6.2 Key points

There are some key features to be considered for modulating the impact of risk and protective factors for depression in young ASD population:

- Comorbid Depression is Common but Underrecognized in ASD Young Population: children and adolescents with ASD face disproportionately high rates of internalizing disorders, particularly depression and anxiety, which often go undetected due to diagnostic overshadowing and communication difficulties.
- Risk Factors Are Multifactorial and Modifiable: key risk factors include low socioeconomic status (SES), lack of social support, sensory overload, and camouflaging behaviors. These factors can be mitigated through structured support and environmental accommodations.
- Lifestyle Interventions Offer Additional Mental Health Benefits: regular physical activity, tailored nutrition (e.g., omega-3s, vitamins B/D), and mind-body practices such as yoga or martial arts are emerging as valuable complementary interventions added to standard care.
- Parent Psychoeducation and Engagement Are Crucial Interventions: they involve caregivers—such as parent training, emotional coaching, and psychoeducation—enhance treatment outcomes and help prevent escalation of subclinical symptoms.
- Early, Personalized Intervention Maximizes Impact: initiating tailored interventions as early as possible, particularly during childhood, may help prevent or mitigate the development and progression of depression and anxiety in autistic children and adolescents.
- Multimodal, Integrated Approaches Are the Future of Care: the synthesis of therapeutic, lifestyle, and social supports in a cohesive care plan provides the most robust defense against mental health deterioration in ASD youth population.

## 7 Conclusions

Children and adolescents with Autism Spectrum Disorder (ASD) are at elevated risk for depression due to a complex interplay of genetic, neurobiological, demographic, psychosocial, and environmental factors. Strengthening protective factors through early diagnosis, family and social support, individualized therapeutic interventions, and healthy lifestyle strategies represents an essential component of comprehensive care. The findings of this narrative overview provide a practical framework for clinicians, rehabilitation specialists, educators, and policymakers to identify modifiable factors associated with depression and implement personalized, multidisciplinary prevention and intervention strategies. Promoting resilience alongside reducing vulnerability has the potential to improve mental health outcomes, quality of life, and long-term functioning in children and adolescents with ASD. Nevertheless, further high-quality longitudinal studies and well-designed randomized controlled trials are needed to strengthen the evidence base and better define the effectiveness of these approaches across the heterogeneous ASD population.

## 8 Future directions

The future research directions proposed in this narrative overview are directly derived from key gaps identified in the current literature. Firstly, the predominance of cross-sectional and observational studies limits causal inference; therefore, longitudinal and randomized controlled trials are needed to clarify the temporal and causal relationships between modifiable factors and depressive outcomes in ASD. Secondly, the marked heterogeneity of ASD populations—with respect to age, cognitive functioning, language abilities, and comorbidities—has not been adequately addressed in existing studies, highlighting the need for stratified and subgroup-specific analyses. Thirdly, the evidence supporting many protective interventions, particularly lifestyle- and rehabilitation-based approaches, remain fragmented and methodologically inconsistent, underscoring the importance of standardized protocols and outcome measures. Finally, while risk and protective factors are often examined separately, there is a lack of integrative models that simultaneously account for both vulnerability and resilience pathways, suggesting a need for transdiagnostic and multidimensional research frameworks. Additionally, future studies should aim to incorporate real-world, ecologically valid outcomes and patient-centered measures to enhance clinical applicability.
