# Supplementary material for: From risk to resilience: a narrative overview of modifiable factors influencing depression in children with autism spectrum disorder (part I)
Source: Front Psychiatry. 2026 Aug 5;17:1859969. doi: 10.3389/fpsyt.2026.1859969 (PMC13487532; doi:10.3389/fpsyt.2026.1859969)
Supplement: Supplementary file 1 [file SupplementaryFile1.pdf]

Supplementary material

Supplementary Table 1. Summary of the Literature Search Strategy

Search Date	Database(s)	Search Terms / Strategy	Filters Applied	Search retrieval counts
17 Apr 2024	PubMed, PMC	Initial exploratory search to identify the breadth of literature related to decreased physical activity level and ASD: "physical activity level" AND "autism" OR "autism"	clinical trial, clinical study, observational study, randomized controlled trial	367
17 Apr 2024	PubMed, PMC	Initial exploratory search to identify the breadth of literature related to physical activity level and ASD: "physical activity level" AND "autism spectrum disorder"	clinical trial, clinical study, observational study, randomized controlled trial	274
17 Apr 2024	PubMed, PMC	Initial exploratory search to identify the breadth of literature related to decreased physical activity level and ASD: "decreased physical activity level" AND "autism spectrum disorder"	clinical trial, clinical study, observational study, randomized controlled trial	46
17 Apr 2024	PubMed, PMC	Initial exploratory search to identify the breadth of literature related to stress, depression, and ASD in young population: "youth" AND "stress" AND "depression" AND "autism spectrum disorder"	clinical trial, clinical study, observational study, randomized controlled trial	9
25 Oct 2024	PubMed, PMC	Initial exploratory search to identify the breadth of literature related to depression and ASD in young population: "youth" AND "stress" AND "depression" AND "autism spectrum disorder"	clinical trial, clinical study, observational study, randomized controlled trial, systematic reviews and meta-analyses	42
25 Oct 2024	PubMed, PMC, Scopus, Google Scholar	Refined search using MeSH terms "autism spectrum disorder" "OR "ASD"AND "anxiety" AND "depression"	clinical trial, clinical study, observational study, randomized controlled trial; systematic reviews and meta-analyses; abstract available	3,487
21 Jun 2025	PubMed, PMC, Scopus, Google Scholar	Further iterative refinement: combinations of "ASD-", "autism spectrum disorder", "depression-", "resilience-", "risk factor-", "protective	Eligibility criteria applied according to narrative review protocol	168

Search Date	Database(s)	Search Terms / Strategy	Filters Applied	Search retrieval counts
		factor-," and "intervention-related" keywords and MeSH terms		
21 Jul 2025	PubMed, PMC, Scopus, Google Scholar	Further iterative refinement: combinations of "ASD-", "autism spectrum disorder", "depression-", "resilience-", "risk factor-", "protective factor-", and "intervention-related" keywords and MeSH terms	Eligibility criteria applied according to narrative review protocol	168
18 Sep 2025	PubMed, PMC, Scopus, Google Scholar	Further iterative refinement: combinations of "ASD-", "autism spectrum disorder-", "depression-", "depressive symptoms-", "internalizing symptoms-", "resilience-", "modifiable risk factor-", "protective factor-", "psychosocial intervention-", "rehabilitation-", and "lifestyle intervention-" related keywords and MeSH terms. Search terms were progressively refined throughout the review process.	Eligibility criteria applied according to narrative review protocol	172
05 Oct 2025	PubMed, PMC, Scopus, Google Scholar	Final refined search: combinations of keywords and MeSH terms related to autism spectrum disorder (ASD), depression, depressive symptoms, mental health, mental health disorders (MHD), resilience, risk factors, protective factors, modifiable factors, prevention, psychosocial interventions, rehabilitation, rehabilitative strategies, sensory regulation, lifestyle interventions, and depression in children and adolescents with ASD. Search terms were progressively refined throughout the review process to optimize the retrieval of clinically relevant and methodologically robust studies.	Inclusion and exclusion criteria applied; duplicate and irrelevant records removed through title, abstract, and full-text screening	Studies contributing to final narrative synthesis (n = 65)

*This narrative overview forms Part I of a two-part series. Initial exploratory searches were conducted to identify literature related to both depression and anxiety in children and adolescents with ASD. Following preliminary evidence mapping, the multidisciplinary team determined that the breadth and heterogeneity of the literature warranted two complementary narrative overviews. Subsequent searches and evidence synthesis were therefore refined according to the specific objectives of Part I (depression) and Part II (anxiety). Searches were conducted iteratively at different stages of the review process and may include overlapping records across databases. In addition, relevant studies were identified through manual screening of reference lists. Consequently, the retrieval

counts presented in Supplementary Table S1 do not correspond directly to the final number of studies included in the narrative synthesis.

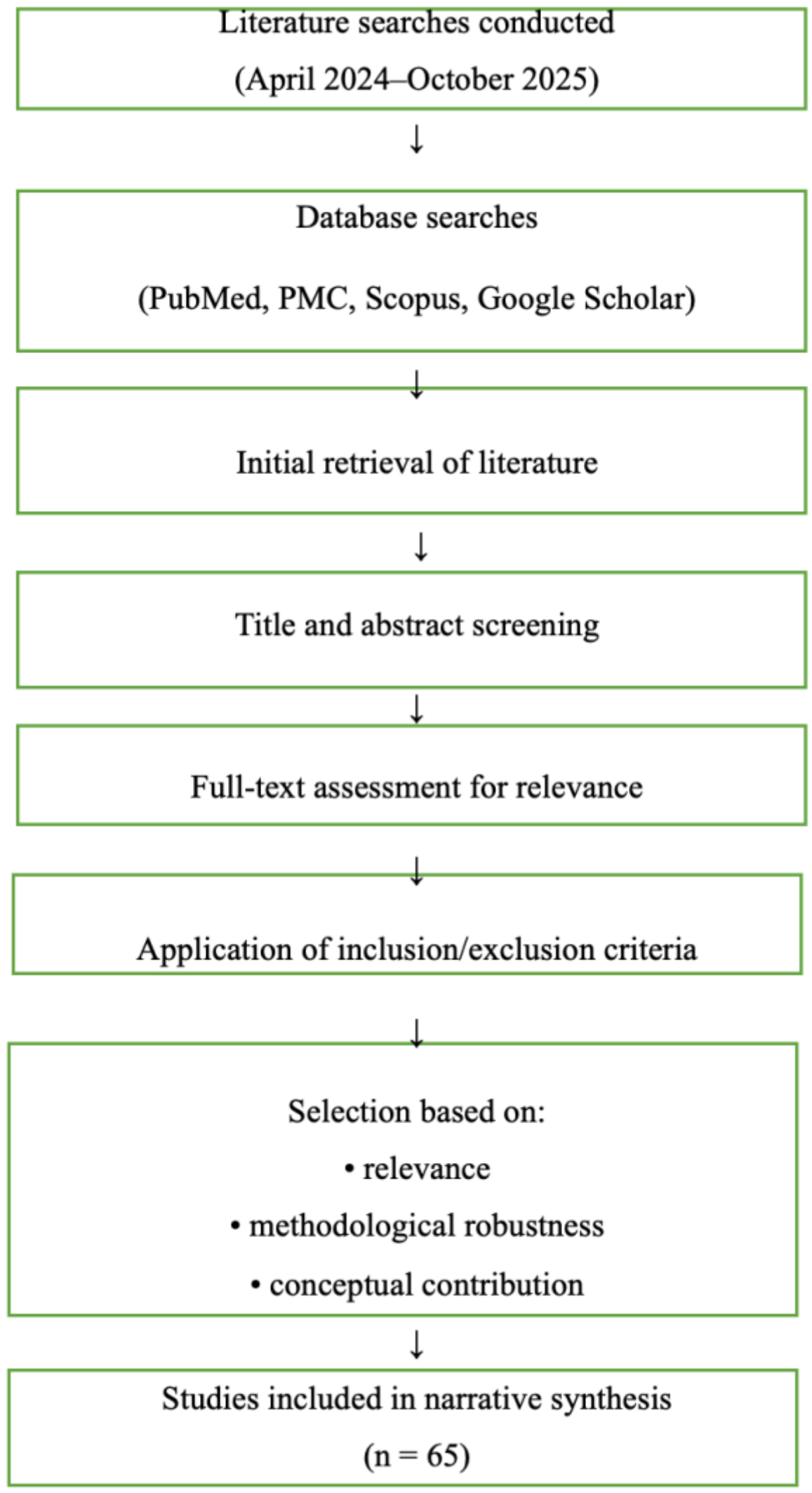

Supplementary Figure 1. Literature identification and study selection process

*Flow diagram illustrating the literature identification and study selection process for this structured narrative overview. Searches were conducted iteratively between April 2024 and October 2025 across PubMed, PubMed Central (PMC), Scopus, and Google Scholar using combinations of ASD-, depression-, and mental health-related keywords and MeSH terms. Final study inclusion was based on predefined eligibility criteria, methodological robustness, and relevance to the objectives of the narrative overview. Evidence from 65 studies was ultimately synthesized.
